# Full Recycling and Re-Use of Bio-Based Epoxy Thermosets: Chemical and Thermomechanical Characterization of the Recycled Matrices

**DOI:** 10.3390/polym14224828

**Published:** 2022-11-09

**Authors:** Sandro Dattilo, Gianluca Cicala, Paolo Maria Riccobene, Concetto Puglisi, Lorena Saitta

**Affiliations:** 1 CNR-IPCB, Via Paolo Gaifami 18, 95126 Catania, Italy; 2 Department of Civil Engineering and Architecture, University of Catania, Viale Andrea Doria 6, 95125 Catania, Italy; 3 INSTM-UDR CT, Viale Andrea Doria 6, 95125 Catania, Italy

**Keywords:** bio-based epoxy resin, chemical recycling, recyclable epoxy, recycled matrices re-use, thermomechanical properties

## Abstract

High performances of thermosets deriving from their covalent intermolecular cross-link bonds result in their low recyclability hindering the full exploitation of a truly circular approach for cured thermosets. In this experimental work, the recyclability of a bio-based fully recyclable epoxy resin using a mild chemical recycling process was demonstrated. The recycled polymer obtained was fully characterized to ascertain its structure and properties. MALDI (Matrix-Assisted Laser Desorption/Ionization), GPC (Gel Permeation Chromatography) and NMR (Nuclear Magnetic Resonance) spectroscopy to determine the chemical structure of the recycled polymer were used. The thermomechanical properties of the cured virgin network and of the recycled product obtained were measured by DSC (Differential Scanning Calorimetry) and DMA (Dynamic Mechanical Analysis). Thermogravimetric analysis of the recycled polymer was also performed. The recycled polymer was transformed into a polyurethane by reacting it with an isocyanate. The synthetized polyurethane obtained therefrom was thoroughly characterized by thermogravimetric analysis. This approach proved the possibility to up-scale the recycled product making it available for novel applications exploiting its re-use.

## 1. Introduction

Epoxy resins are considered as one of the most commercialized classes of thermosets. Several advantages, such as excellent thermal and mechanical properties, easy processability, and low production costs, make epoxy resin thermosets suitable for a wide range of applications. Following their huge commercial production, for applications in, for example, the automotive and aerospace industries, in 2019 the global epoxy market reached 26 billion dollars with an expected increase at a compound annual growth rate (CAGr) of 6.2% from 2020 to 2028 [1]. Epoxy resin is rapidly beginning to replace conventional materials including coatings, protective films, and structural composites. Notwithstanding their excellent properties and evidently wide markets, a crucial drawback still remains concerning their non-recyclability, especially due to their crosslinked nature and the popularity of their combination with other materials (metal fixings, carbon fiber, hybrid composites) [2]. The low recyclability of epoxy resins is considered their most critical issue due to the difficulty in reprocessing the waste obtained at the end of their life or as a by-product from production. With epoxy resin being a permanent cross-linked polymer, it cannot be fused, solubilized, or re-processed as thermoplastics can be; therefore, once the end of life has been reached, just two major alternatives currently exist: landfill or incineration [3]. However, environmental legislation is becoming more and more restrictive, and the environmental impact of these materials when disposed in landfill is accelerating the urgency to reach an industrial-scale method for the recycling of composites reinforced with epoxy resin [4]. In the near future, landfill and incineration will become increasingly restricted or could be banned, driving industries and their customers to look for more sustainable solutions, according to the European Composites Industry Association (EuCIA) [5]. The increasing cost to dispose of such materials in landfill is an additional factor pushing industries to find recycling strategies for thermosets. This background explains the growing need to find more environmentally friendly solutions for the treatment of thermosets. The need for a suitable recycling approach for thermosets is particularly relevant for composites reinforced with carbon fiber (CF), manufactured with epoxy matrices. In fact, in this case the presence of expensive and environmentally impacting carbon fibers is a further reason pushing industries to develop recycling strategies. Moreover, social and economic pressure to develop the greenest alternative manufacturing methods, as well as the increasing use of Life Cycle Analysis (LCA) [6], further promotes the challenge to develop the most sustainable, least toxic, and most recyclable epoxy resin. For the reasons discussed above, many different recycling techniques have been developed for CF-reinforced composites with an epoxy matrix [7,8]. Currently, chemical, mechanical, and thermal treatments have been proposed as the principal recycling procedures for carbon-fiber-reinforced composites. Among all these procedures, the chemical process has received much attention due to the easy separation of each component, with the possibility of recovering the carbon fibers mostly unaltered [9,10]. Solvolysis, as one of the most studied chemical recycling procedures, is based on the combination of solvents and a catalyst triggering the depolymerization of the matrix with the recovery of the undamaged fibers [10,11,12,13,14,15]. Solvents such as supercritical fluids [16,17,18,19] and alcohols [19,20,21,22,23] are commonly used to penetrate across the composite and promote bonding dissociation of a thermoset such as an epoxy matrix. Due to the varying nature of thermosets (epoxy resin, polyester [24,25,26,27], cyanate ester, phenolic resin, polyamides, etc.) and fillers (carbon fiber, glass fiber, ceramics, metals, etc.), chemical recycling offers broad possibilities thanks to the huge range of combinations of solvents, catalysts, and temperatures [13,16,28,29,30,31,32]. However, a limitation of this approach is that the depolymerized matrix obtained is not re-usable in a straightforward approach due to the structure of the products obtained. Simple monomers and mixtures are usually derived with standard solvolysis requiring further purification and complex synthesis to obtain a usable matrix [33,34]. Although there are several works in the literature on the chemical recycling of thermosets, it remains a relatively new area, with the challenge to obtain a scalable process for industrial application remaining.

In this work, a chemical recycling process for epoxy resin matrices was implemented starting from a procedure reported previously [35,36] and making significant changes to obtain a more environmentally friendly procedure. An almost complete recovery of the used chemical solvents was achieved, thus resulting in a lack of chemical waste that otherwise would have caused a negative impact on the environment. Thus, since the main drawback related to chemical recycling as a disposal route for epoxy resins is that some used chemical solvents can be toxic to the environment [1], the novel proposed procedure ensures the avoidance of this issue. This feature is crucial for developing a novel, truly green recycling approach for epoxy-based composites [37]. The procedure is based on a special amine, which presents a cleavable ketal group within its structure, enabling the selective cleavage of the cured network under mild acidic conditions. At the end of the process, starting from a totally cured thermoset material, a recycled polymer (with a thermoplastic nature) was obtained. The first part of the study is focused on the optimization of the chemical recycling process described so far, and on the chemical and thermo-mechanical characterization of the recycled polymer obtained from the same. Even though a chemical structure for the recycled polymer (rTP) obtained from the recycling process was hypothesized in a previous study [35], its chemical structure was never experimentally determined. Next, with the rTP’s chemical structure fully determined through matrix-assisted laser desorption/ionization mass spectroscopy (MALDI–MS), our re-use strategy was determined by following a *‘design from recycling approach’* to accomplish a circular economy approach. In detail, with the rTP classifiable as a polyol, a polyurethane (PU) was synthetized starting from the rTP to prove the up-scaling strategy of our approach. This choice is justified by the fact that PUs are commonly synthetized starting from a compound characterized by two or more hydroxyl groups (-OH) and isocyanates. Furthermore, the production of this group of polymers starting from a green raw material (i.e., recycled polymer) rather than a petroleum oil-based polyol [38] is pivotal to synthetizing a more environmentally friendly PU. This approach represents an interesting novel achievement, since PUs are suitable for a wide range of applications and might reveal a wide range of properties [39,40,41].

## 2. Materials and Methods

### 2.1. Materials

A bio-based epoxy resin system was used as a matrix for the study. The resin is a bicomponent system obtained by mixing a bio-based epoxy prepolymer named Polar Bear (part A) and the amine hardener Recyclamine^TM^ R*101 (part B). The two components were purchased from R*CONCEPT (Barcelona, Spain). The epoxy prepolymer Polar Bear was characterized using the standard ISO/IEC 17025:2017 PJLA #59423 and, according to the results, the biocarbon content is equal to 28% (expressed as a fraction of the total organic carbon content). The hardener Recyclamine^TM^ R*101 is the reactant that, bearing the cleavable ketal group, led to the full recyclability of the cured resin system. Thus, these cleavable groups in the cross-linked network are the key points that can be broken under the mild acidic bath used for the recycling. Both the part A (Polar Bear) and the part B (curing agent Recyclamine^TM^ R*101) are liquid at 25 °C, hence favoring the mixing of the epoxy formulation.

The reactants for the chemical recycling of epoxy resin and for the synthesis of Polyurethane are listed below: pure acetic acid from VWR International S.r.l., Milan, Italy; ammonium hydroxide (28.0–30.0% NH_3_ basis) from Sigma-Aldrich (Merk Life Science S.r.l., Milan, Italy); 1,6-Diisocyanatohexane 98% from Sigma Sigma-Aldrich (Merk Life Science S.r.l., Milan, Italy); stabilized Tetrahydrofuran ≥99.5% (by anhydrous basis) AnalaR NORMAPUR^®^ ACS, Reag. from VWR Chemicals (VWR International S.r.l., Milan, Italy).

### 2.2. Epoxy Resin Formulation

Resin formulations were produced by mechanical mixing of part A and part B. The amount of the latter was equal to 22 phr (per hundred resin). All the mixtures were degassed, during mixing, at room temperature using a planetary mixer with vacuum conditions (Thinky mixer ARV310, THINKY U.S.A. Laguna Hills), which is characterized by a maximum mixing volume of 300 mL, then poured in silicon molds to manufacture samples (in the form of bars) with a size of 80 mm × 10 mm × 4 mm, and cured for 24 h at room temperature (i.e., 25 °C). These samples were named cured (E1). The room temperature curing was followed by a post-cure process at 100 °C for 3 h in a static oven and the samples obtained therefrom named post-cured (E2). Both the cured and post-cured samples were then recycled to understand the effect of the curing cycle on the chemical recycling process.

All the information about the two investigated epoxy resin systems investigated, which differ each other in the curing cycle, are summarized in Table 1.

### 2.3. Chemical Recycling Process

The two cured epoxy matrices were recycled using a chemical recycling process. This approach was initially developed and presented by Connora^®^ Technologies [36,42]. In this study, a more environmentally friendly process is proposed in which 85% of the chemical aqueous solution used for the cleavage process of the epoxy matrix was recovered, a smaller consumption of the ammonia solution used for the neutralization procedure achieved, and the optimization of the drying phase was performed, thus reducing energy consumption.

The procedure for the chemical recycling is described in the following: 10 g of cured epoxy resin was dissolved in 300 mL of acetic acid aqueous solution (75% *v*/*v*) at 80 °C for 90 min. Subsequently, the solution obtained was concentrated under vacuum (60 mbar of pressure) by roto-evaporation at 60 °C up to a volume of 75 mL. The precipitation of the cleaved epoxy resin was obtained by neutralization with 150 mL of ammonium hydroxide solution prepared by mixing water and ammonium hydroxide (28–30% NH_3_ basis) in a 1:1 ratio. Once the precipitation step was completed, the precipitated solid residue was filtered, washed with deionized water, and dried under vacuum at 50 °C for 24 h. Finally, the dried residue was pulverized by using a mortar and pestle and characterized.

The evaporated water solution obtained in the roto-evaporation step was collected and showed a volume of about 85% of the initial acetic acid solution. This solution was stored and re-used for further recycling processes. Moreover, because of the roto-evaporation, the amount of solution used for the neutralization step was 1/3 of the amount used for the Connora^®^ process. Hence, the new recycling procedure made it possible to reduce environmental impacts.

**Figure 1:**
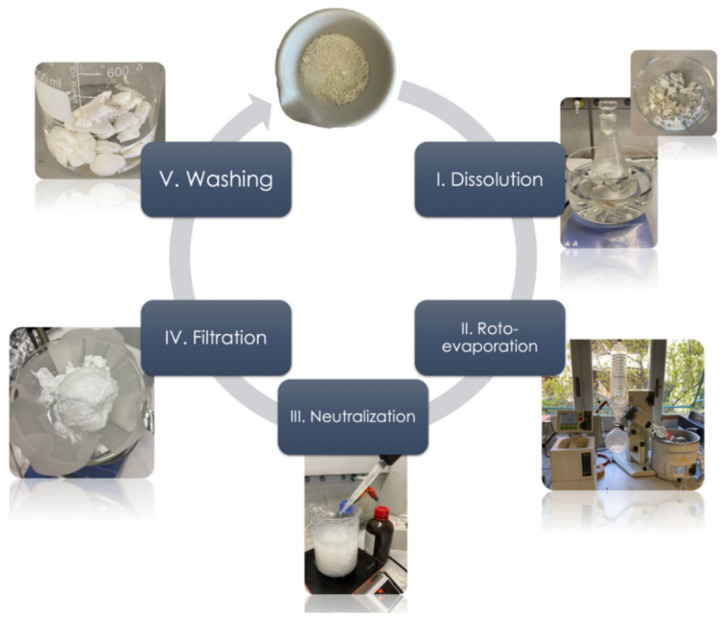
Main steps of the chemical recycling process.

The main steps of the recycling process are depicted in Figure 1.

### 2.4. Re-Use and Up-Scaling Strategy of the Recycled Polymer

The recycled polymer (rTP), which has the nature of a thermoplastic, was thoroughly characterized and its properties up-scaled using a synthetic strategy based on reaction with 1,6-Diisocyanatohexane to obtain a polyurethane. The synthetic procedure was the following: 50 mg (about 5 mmol) of the recycled thermoplastic was solubilized in tetrahydrofuran (THF); after that, to the latter solution were added 15 mg (about 89 mmol) of 1,6-Diisocyanatohexane; next, the temperature was raised at 60 °C for four hours. At the end of the reaction, a whitish precipitate appeared within the solution, which was the synthetized polyurethane. In detail, the reaction underlying the synthesis of this material is the bond between the functional group (-NCO) of the 1,6-Diisocyanatohexane and the groups (-OH) of the recycled thermoplastic, which were identified through MALDI analysis.

### 2.5. Characterization Techniques

#### 2.5.1. Dynamic Mechanical Analysis (DMA) of the Cured Epoxy Resins

To determine the glass transition temperature (Tg) of the epoxy resin cured with different curing cycles (E1 and E2), dynamic mechanical analysis was carried out. The dynamic mechanical thermal analyzer TRITEC 2000 (Triton Technology, Leicestershire, UK) was used for testing. The DMA specimens had a size equal to (10 × 6 × 4) mm^3^. A single cantilever deformation mode was used, as it is recognized as a performing mode for characterization through the glass transition [43]. The specimens were heated from RT up to 150 °C with a heating ramp rate of 2 °C/min. For each test, the displacement was set at 200 μm. The tan δ versus temperature at 1 Hz was plotted for the two curing cycles investigated. Three samples were tested for each cross-linking cycle implemented; next, the average and the standard deviation were evaluated for the Tg. A comparison between the two responses taken into consideration (Tg value for E1 and E2) for the two different curing cycles analyzed was carried out by means of a pooled *t*-test.

#### 2.5.2. Gel Permeation Chromatography (GPC)

A SEC system consisting of an integrated Waters 515 apparatus (a pump and an injector) and a Waters R401 DRI detector, using THF as the mobile phase (1 mL min^−1^ flow rate), was used for determining the molecular weight (MW) of the recycled polymers. The column set was composed of four Ultrastyragel HR columns (ID = 7.8 mm, L = 300 mm, 5 μm particle size) in the order HR-4, HR-3, HR-2, and HR-1 connected in series. The SEC traces were recorded and processed using the *Clarity-GPC* software provided by *DataApex*. In a typical analysis, 100 μL of a polymer solution in THF (ca. 5 mg mL^−1^ concentration) with 2 μL of dichlorobenzene added was used as flow marker.

#### 2.5.3. Matrix-Assisted Laser Desorption/Ionization Time-of-Flight Mass Spectrometry (MALDI-TOF-MS)

In MALDI-TOF-MS analysis, a dilute solution of the analyte polymer (2 mg/mL) was mixed with a more concentrated matrix solution (20 mg/mL), and the ‘dried droplet’ method was utilized for sample preparation. Mass spectra were recorded using trans-2-[3-(4-tert-butylphenyl)-2-methyl-2-propenylidene]malononitrile (DCTB) as a matrix. MALDI-TOF mass spectra were recorded in linear mode by means of a 4800 Proteomic Analyzer (Applied Biosystems) MALDI-TOF/TOF instrument equipped with an Nd:YAG laser at a wavelength of 355 nm with <500 ps pulse and 200 Hz firing rate. The accelerating voltage was 15 kV. External calibration was performed using an Applied Biosystems calibration mixture consisting of polypeptides with different molar mass values. The irradiance was maintained slightly above the threshold to obtain a mass resolution of about 7000–9000 fwhm. Mass accuracy was about 50 ppm.

#### 2.5.4. Heated Electrospray Ionization Mass Spectroscopy (HESI-MS)

ESI mass spectra were acquired by a Thermo Scientific Exactive Plus Orbitra MS (Thermo Fischer Scientific, San Jose, CA, USA), using a heated electrospray ionization (HESI II) interface. Mass spectra were recorded operating in positive ion mode in the m/z range of 150–1000 at a resolving power of 25,000 (full-width-at-half-maximum, at m/z = 200, RFWHM, and a C-trap inject time of 100 ms) under the following conditions: capillary temperature 275 °C; nebulizer gas (nitrogen) with a flow rate of 5 arbitrary units; auxiliary gas flow rate of 1 arbitrary unit; source voltage equal to 3.5 kV; capillary voltage of 82.5 V; tube lens voltage of 120 V. The Orbitrap MS system was tuned and calibrated in positive modes, by infusion of solutions of a standard mixture of caffeine (Mr 194.1 Da), MRFA peptide (Mr 423.6 Da), and Ultramark (Mr 1621 Da). Data acquisition and analysis were performed using the *Excalibur* software.

#### 2.5.5. Proton Nuclear Magnetic Resonance (^1^H-NMR) Analysis

The ^1^H-NMR spectra of the monomer utilized for the epoxy resin synthesis were obtained by using a Bruker Advance 400 spectrometer (Milan, Italy). To run the analyses, which were performed at 40.013 MHz, the samples were dissolved in deuterated chloroform (CDCl_3_) at the concentration of 10 mg/mL. The chemical shifts were measured and the assignments of the protons were accomplished.

#### 2.5.6. Thermal Gravimetric Analysis (TGA)

The thermal stability for the recycled polymer and the polyurethane synthetized as the re-use strategy was investigated by TGA (TA Instruments Q500, New Castle, DE, USA), under nitrogen flow (60 mL/min), at a heating rate of 10 °C/min, from 50 to 800 °C, using 2–5 mg of sample. Instrument temperature and weight were calibrated by using high-purity magnetic standard for the Curie temperature and some exact weights, respectively, following the calibration procedure by the instrument control software. The TGA data were analyzed by the TA Instruments operating software (New Castle, DE, USA). For each sample, weight loss versus temperature was determined.

#### 2.5.7. Differential Scanning Calorimetry (DSC)

A Shimadzu DSC-60 (Shimadzu, Kyoto, Japan) device was used to carry out the calorimetric measurements. The DSC analyses were run for the recycled thermoplastic obtained from the chemical recycling process to determine the Tg value. For each analysis carried out, about 6 mg of sample was put into 40 μL sealed aluminum crucibles. Once the analysis started, each sample was heated from 25 °C up to 250 °C at a rate of 20 °C/min in air.

#### 2.5.8. FT-IR Analysis

The Fourier Transform Infrared Spectroscopy (FT-IR) spectra of both epoxy resin (E2) and recycled polymer (rTP) were recorded using a *PerkiElmer Spectrum 100 UATR* (Waltham, MA, USA) spectrometer in attenuated total reflectance (ATR) mode. The absorption bands were recorded in the range of 4000−650 cm^−1^ with 16 scans and a resolution of 4 cm^−1^. The data were analysed using *OMNIC* software. This analysis allowed to investigate how the rTP structure changed, starting from the initial epoxy system from which it derives.

## 3. Results and Discussion

### 3.1. Thermo-Mechanical Properties of Cured Resins

**Figure 2:**
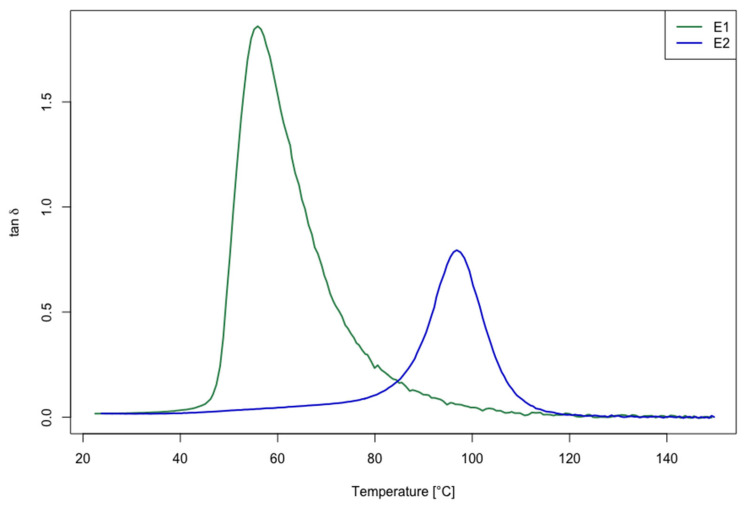
DMA results: tan δ versus temperature plot for E1 (green curve) and E2 (blue curve) epoxy systems.

The results obtained for Tg from the DMA are reported in Table 2, and the tan δ versus temperature are depicted in Figure 2. The DMA analysis clearly shows the tan δ peak shift to a higher temperature for the E2 samples which, for the post-cured samples, is also lower in height.

The difference of the Tg mean values for the two considered curing cycles (E1 and E2) is statistically significant (*p*-value * < 0.05). Therefore, the E2 formulation has a significantly higher Tg (of about 72%) compared with the E1 epoxy matrix. The shift of the Tg value toward a higher temperature is due to the higher crosslink density obtained by increasing the curing temperature [44]. For this reason, it is important to study the effect of the recycling process on the cured and post-cured samples that, as shown from DMA, presented a different cured network density.

### 3.2. Chemical Recycling Process Results and Yields

The recycled polymers obtained from the epoxy resin systems cured at two different curing cycles were identified as rTP–I (derived from E1) and rTP–II (derived from E2). The recycled polymers were both whitish solids that could be easily crushed to form a powder by using a mortar and pestle, as shown at the end of the process cycle in Figure 1. The yield of the chemical recycling process was calculated by considering the formula below:

(1) $$yield\;\left(\%\right)=\frac{W_{i}^{\left(E\right)}}{W_{f}^{\left(rTP\right)}}\times 100$$

with $W_{i}^{\left(E\right)}$ being the initial weight of the epoxy resin recycled and $W_{f}^{\left(rTP\right)}$ the final weight of the recycled thermoplastic (after being dried) obtained from the implemented chemical recycling process. It was equal to 99% both for rTP–I and rTP–II. Therefore, despite the two cured samples having different cured densities, the recycling conditions used allowed the full depolymerization of the cured network, yielding the full recovery of the matrix.

### 3.3. Chemical Characterization of the Recycled Polymer

**Figure 3:**
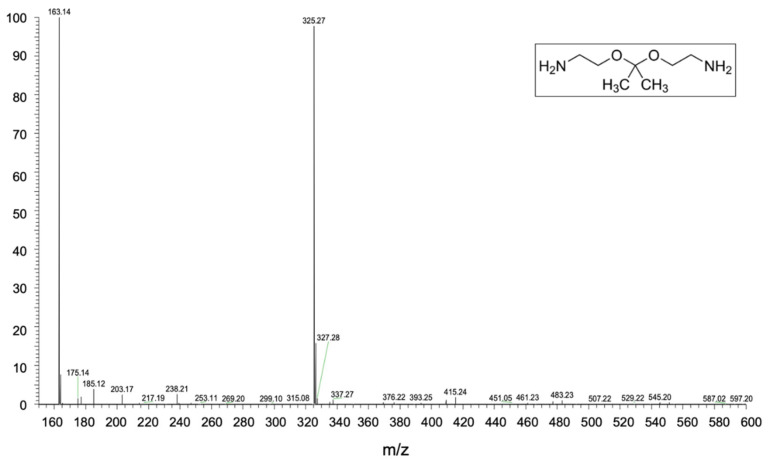
HESI-MS spectra of Recyclamine^TM^ cross-linked agent.

In order to explain the structure of the polymer obtained from the recycling process, different types of analyses were carried out. HESI-MS analysis of the amine used as a cross-linking agent was carried out first. As can be seen from the spectrum shown in Figure 3, there are two peaks at m/z equal to 163.14 and 325.27 corresponding to 2,2-Bis (aminoethoxy) propane and its dimer as a protonate adduct. Consequently, the chemical structure of the amine used is the one reported in the inset of Figure 3 which corresponds to Recyclamine^TM^ R*101 (part B).

**Figure 4:**
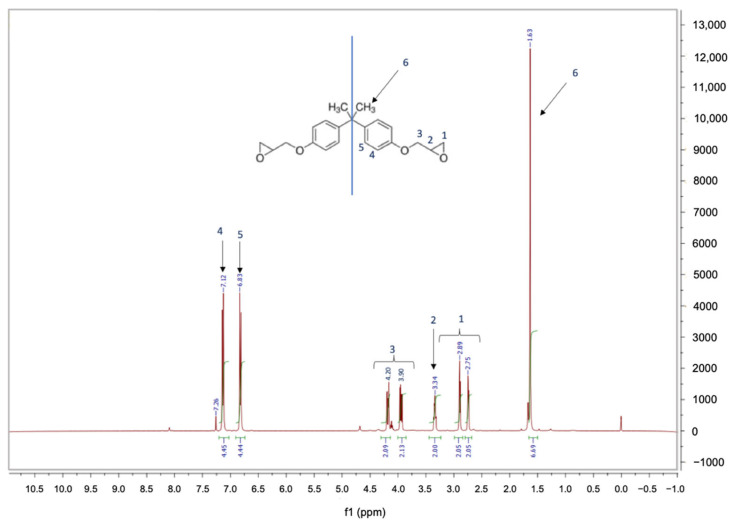
^1^H-NMR spectra of the monomer Recyclamine^TM^ R*101.

The **^1^**H-NMR analysis of the monomer Recyclamine^TM^ R*101 (part B) was carried out to be helpful for the interpretation of the cross-linked resin’s MALDI-TOF-MS analysis once the acid cleavage occurred. The acquired spectra and the proton assignments are shown in Figure 4. The obtained **^1^**H-NMR spectra correspond to a bisphenol A diglycidyl ether ‘DGEBA’ monomer one [45]. In fact, the molecule shows a plane of symmetry and a signal with a chemical shift of 1.63 (6) ppm, which is due to six protons of the methyl group of bisphenol A. The signal of protons which resonate in the range between 2.75–4.20 (1,2,3) is due to ten protons of the aliphatic chain linked to oxygen, whereas the eight aromatic protons of bisphenol A unit give two doublets at 6.83 (5) and 7.12 (4) ppm.

**Figure 5:**
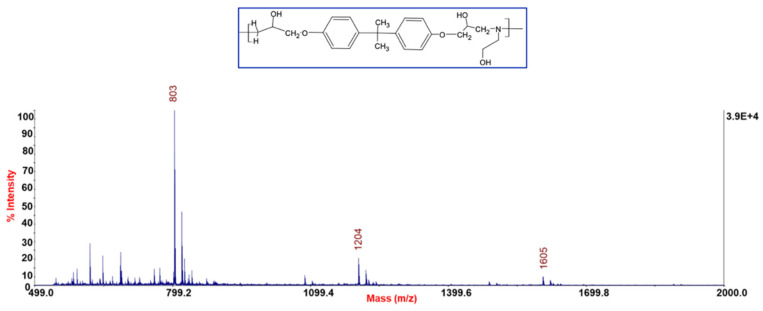
MALDI-MS spectra of rTP recycled polymer. Within the blue box is shown the corresponding repetitive unit’s chemical structure.

**Figure 6:**
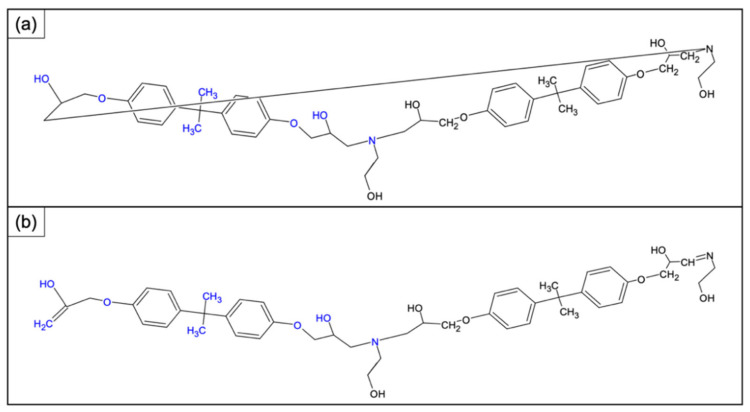
**Dimeric** structure with m/z 802 determined once the repetitive unit is known: hypothesized (**a**) cyclic structure and (**b**) linear structure with ending unsaturation points.

The resin cross-linked with this amine was subjected to cleavage with acetic acid. The selective cleavage occurred at the ethereal bridges with the formation of a polymeric material which was characterized by MALDI-TOF-MS (Figure 5). MALDI-TOF mass spectra give detailed information about the chemical structure of polymers, allowing us to look at the mass of an individual molecule in a mixture of homologues, and permitting the structural identification of a single macromolecular chain (linear and cycles). Often, MALDI-TOF mass spectra allow the identification of repeat units, end chains, cyclic oligomers, and species present in minor amounts. MALDI-TOF mass spectra of all samples recorded in reflectron mode showed mass peaks in the mass range m/z 800–3000, permitting assignments to the corresponding oligomers. In Figure 5 it is possible to see families relative to protonate adducts of peaks spaced from 401 m/z corresponding to a repetitive unit of the rTP recycled polymer (i.e., between 803 and 1204 etc. m/z highlighted in the chart). Figure 6 shows two possible dimeric structures with m/z = 802; among them the linear one is the most probable due to the epoxy resin matrix.

**Figure 7:**
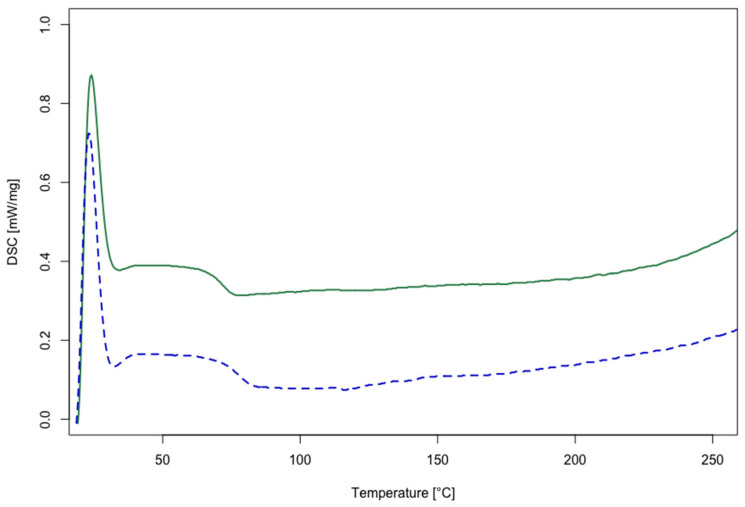
DSC thermograms of the recycled thermoplastics obtained from the chemical recycling process: rTP–I (green curve) and rTP–II (blue curve).

The recycled polymers obtained (rTP–I and rTP–II) were characterized by DSC analysis to determine their Tg. The results obtained are shown in Figure 7. The rTP–I showed a Tg value of 71 °C, whereas for the rTP–II it was equal to 76 °C. Thus, the post-curing process, added for the initial epoxy resin (E2), allowed us to obtain a recycled polymer with a slightly higher Tg, i.e. an increase of 7%, compared with the recycled polymer obtained from E1 recycling. Similar values were reported previously for recycled polymers derived from other bio-based epoxy resins cured with cleavable amines [42,46].

**Figure 8:**
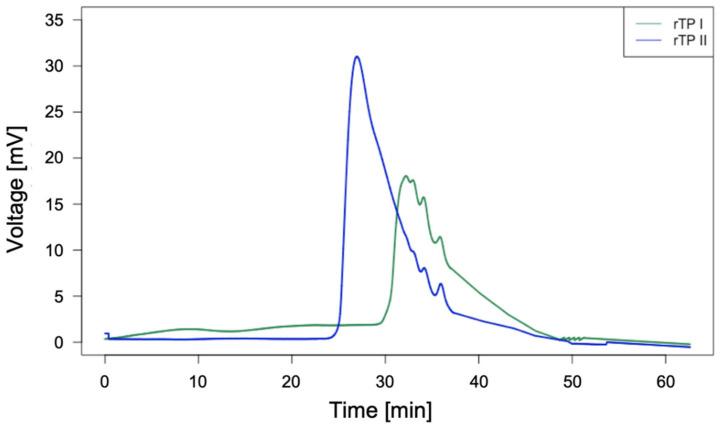
GPC results for the recycled thermoplastic obtained using the E1 epoxy system (green curve) and the one obtained from the E2 epoxy system (blue curve).

Next, the GPC analysis was carried out to determine the molecular weight of the rTP samples. From the results, it can be stated that the MW values of the recycled thermoplastic rTP–I and rTP–II obtained are approximately 9000 Da and 15,000 Da, respectively. Therefore, it was possible to obtain an increase in molecular weight of about 40%. In Figure 8 it is possible to observe a comparison between the chromatograms related to the two recycled polymers obtained starting from two initial resin systems which are identical in composition, but differ for the implemented curing cycle.

According with the results shown by the GPC analysis, it is possible to assess that the chosen curing cycle influenced both the thermal properties (i.e. Tg) and the molecular weight of the recycled polymer produced. The higher the Tg of the recycled epoxy resin, the better the quality of the recycled product in terms of molecular weight. It is proven by the fact that an increase of 72% for the Tg value of the initial epoxy resin is accompanied by an increase of about 67% for the molecular weight of the recycled product. This result can be explained through the Flory–Fox equation, which relates the average molecular weight of a polymer to its glass transition temperature [47].

**Figure 9:**
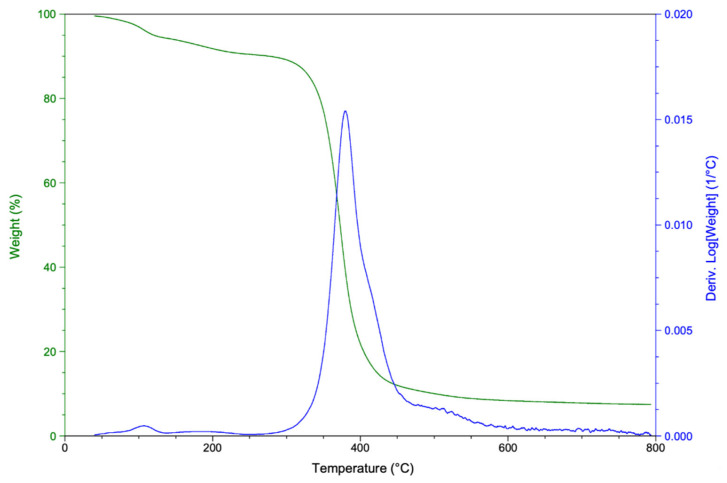
TGA (green curve) and DTG (blue curve) thermograms of rTP–II.

Figure 9 shows the TGA thermogram of rTP–II. Similar findings were obtained for rTP–I. The curing cycle had no significant effect on the thermal stability of the recycled polymer. Thermal decomposition of the recycled thermoplastic rTP–II mainly took place in one region. The maximum degradation rate is about 400 °C as reported for degradation of DGEBA-based epoxy resins under inert atmosphere [48] due to a chain scission of the C–O bond. However, the initial mass loss (about 10%), which is dominated by moisture release, begins around 50 °C. Moistures were, at first, absorbed during the chemical recycling process, and then remained trapped in the recycled thermoplastic’s structure during the drying phase.

**Figure 10:**
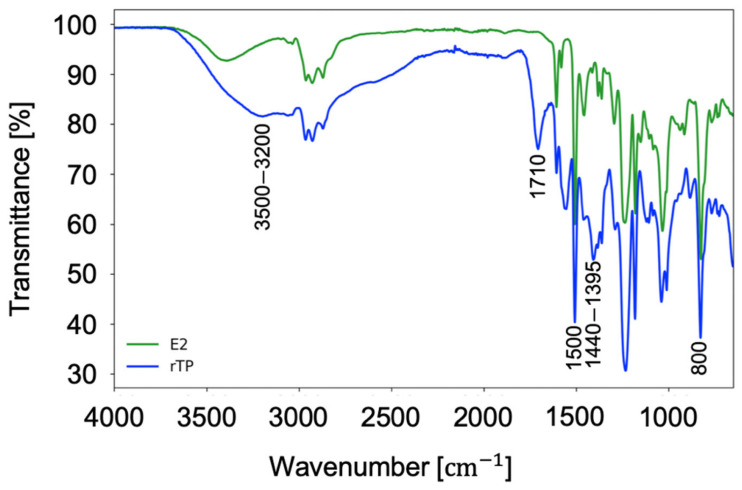
FT-IR acquired spectra for the initial epoxy resin (green curve) and the recycled polymer (blue curve).

Finally, Figure 10 shows the acquired spectra for the FT-IR analysis carried out. First, focusing on the spectrum related to the initial epoxy system (green curve), the total absence of the characteristic peak corresponding to the oxirane groups (910–810 cm^−1^) and the simultaneous presence of broad band related to the stretching of OH bonds (3500–3200 cm^−1^) must be highlighted, which demonstrates the completed cross-linking process of the material. Furthermore, the other characteristic bands are related to the intense vibrations of aryl-alkyl-ether groups (1250 to 1170 cm^−1^), the vibration of aromatic rings (1500 cm^−1^), and 1,4-disubstituted aromatic rings (800 cm^−1^). Whilst, moving on the results acquired for the recycled polymer (rTP), a similar spectrum was obtained. This result is justified by the cleavable process mechanism occurring only in the correspondence of the cross-link cleavable points, by keeping the chemical structure between them unchanged.

### 3.4. Re-Use and Up-Scaling Strategy: Polyurethane Synthesis

**Figure 11:**
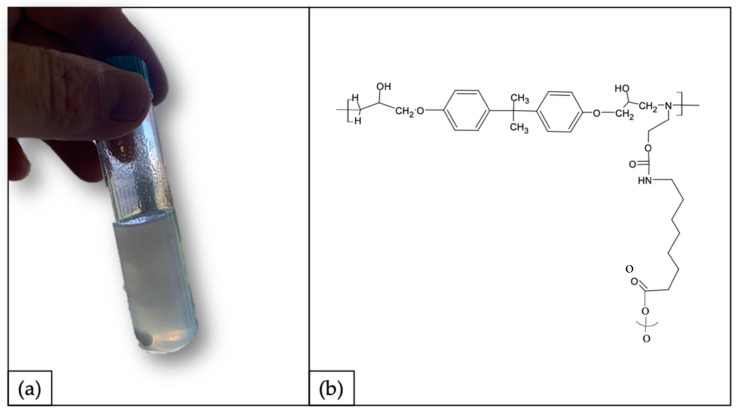
(**a**) Synthetized polyurethane as re-use strategy: proof of concept and (**b**) hypothesized chemical structure for the synthetized polyurethane.

By following a circular approach with no waste production, as a proof of concept, the recycled polymer was used to synthetize a polyurethane (PU) matrix. In accordance with the process described in Section 2.4, the synthetized PU is shown in Figure 11a. The material obtained appeared softer and less brittle compared with rTP–I and rTP–II. This result can be explained as the outcome of the synthesis of a branched structure embedding the isocyanate chains (shown in Figure 11b).

**Figure 12:**
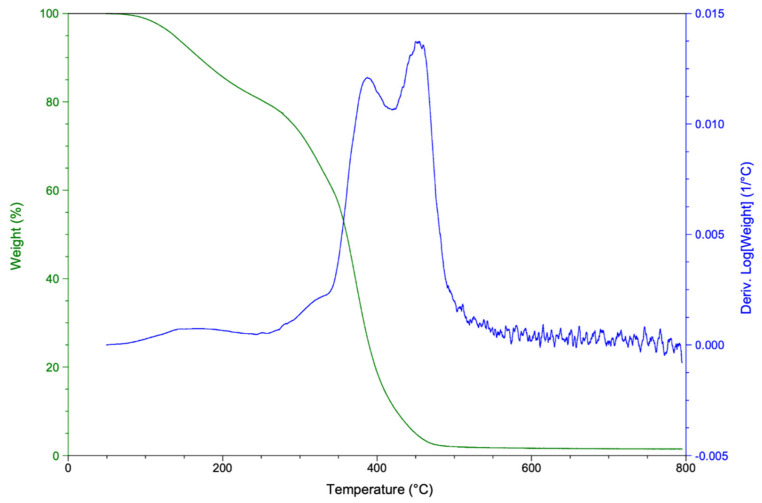
TGA (green curve) and DTG (blue curve) thermograms for the synthetized polyurethane.

The thermal degradation for the polyurethane produced starting from rTP–II was investigated. Both the obtained TGA and DTG thermograms are shown in Figure 12.

According to the thermogravimetric curves acquired, the synthetized PU mass loss, i.e. thermal degradation, started at around 200 °C. This behavior is likely due to the breaking of the C–O bond in hard segment carbamate, which is decomposed in isocyanate and polyol. Next, the PU became fully thermally degraded at about 450 °C. A similar result has been identified by K. Ismoilov et al. [49], who synthetized a waterborne polyurethane finishing agent.

## 4. Conclusions

A bio-based fully recyclable epoxy resin cured by different curing cycles was characterized using dynamic mechanical analysis. Next, its recyclability was proven through an optimized chemical recycling process leading to the recovery (i.e., 99%) of a reusable polymer from the EoL-cured epoxy. The optimized recycling procedure limited environmental impacts, such as through the recovery of the acetic acid solution and the re-use of the ammonium hydroxide solution for at least three recycling cycles. These savings and re-use of solvents and reagents are critical in view of the development of a truly green recycling approach for epoxy-based composites. These findings represent a clear advantage over the previously reported chemical recycling approach used for similar resin systems [36,42]. The exact evaluation of the avoided impact by using the proposed optimized chemical recycling procedure is currently under study using an LCA approach that will be published in a forthcoming paper. However, according with previous LCA and LCC (Life Cycle Cost) studies already proposed by A.D. La Rosa et al. [50] for the similar precursor (not optimized) recycling approach proposed by Connora^®^ Technologies, the selection of this disposal route for the *closed loop* recycling of epoxy-based carbon fiber reinforced composites allows to achieve both a significant reduction in terms of global warming potential (GWP) and a considerable reduction of costs from EUR 288 down to EUR 2.91 for about 35 kg of composite material. The latter benefits are expected to mainly come from the recovery of CFs, which are very expensive raw materials (estimated cost for 2020 of USD 5–20/m^2^) [7]. Similar results have been obtained by considering an *open loop* scenario, where even avoided costs related to the recycled thermoplastic were accounted for. Starting from the encouraging results, the recycling approach proposed in this experimental work should enable even greater benefits in terms of environmentally avoided impacts and economic benefits. Indeed, a remarkable recovery rate for chemicals used for the recycling process was achieved together with a reduction of energy consumption related to an optimization of the drying phase.

In this paper, the recycled polymer obtained starting from the cured epoxy was thoroughly characterized by means of TGA and chemical analyses. Two chemical structures were found for the recycled polymer as a result of the selective bond cleavage of the cured network resulting from the chemical recycling. The recycled polymers obtained resembled the structures of the poly (hydroxyaminoethers) with a thermal stability up to 400 °C [51]. A clear correlation was observed between the cure degree of the epoxy resin and the molecular mass of the recycled polymer obtained therefrom. When post-cured epoxy resin was recycled, a polymer with a molecular mass of about 15,000 Da was obtained. This value was 40% higher than those of the recycled polymer obtained from the room temperature-cured resins. This finding is relevant for applications because it confirms that better properties are obtained by treating cured networks to a higher curing degree. The latter are the epoxy resins which, because of their thermal resistance, usually find applications in the automotive and naval sectors. In fact, using a cured network with a Tg of about 100 °C is the standard in those fields. Furthermore, since existing automotive sector specific EU regulations require the recycling of at least 85% of end-of-life materials [52] together with the CO_2_ emission threshold values regulated by the international regulatory bodies (i.e. 95 g CO_2_/km for cars for the period 2020–2024) for cars, light commercial vehicles, and ship transports for either goods or passengers, the use of lightweight bio-based fully recyclable epoxy composites might be a highly profitable alternative. Indeed, the usage of this class of material ensures both the reduction of paying load and the conversion of waste (reinforcement fibers and epoxy matrix) into a valuable reusable resource, thus satisfying a circular economy approach.

Finally, as an important outcome of the study, the successful synthesis of a polyurethane using a recycled polymer as a building block was reported. The synthesis described in the paper turned the recycled material from a brittle to a soft solid. This finding can pave the way to an up-scaling strategy for the recycled polymer, opening an undisclosed field of applications such as, for example, dampeners or foams. More research will be needed to further optimize the synthetic strategy, for example, using bio-based isocyanates as reactants and optimizing the reaction conditions to make them readily available for standard PU processing.

## Figures and Tables

**Table 1:** Epoxy resin systems analyzed.

Sample ID	Formulation	Mixing Ratio by Weight	Curing Cycle
E1	Polar Bear + Recyclamine^TM^ R*101	100:22	at 25 °C for 24 h
E2	Polar Bear + Recyclamine^TM^ R*101	100:22	at 25 °C for 24 h + 100 °C for 3 h

**Table 2:** Tg results obtained from DMA for the two investigated curing cycles (E1 vs. E2).

Sample ID	Tg [°C]
E1	60.30 ± 0.26
E2	103.40 ± 0.20

## Data Availability

No data supporting reported results exists.
